# Automated classification of pollen grains microscopic images using cognitive attention based on human Two Visual Streams Hypothesis

**DOI:** 10.1371/journal.pone.0309674

**Published:** 2024-11-21

**Authors:** Mohammad Zolfaghari, Hedieh Sajedi

**Affiliations:** 1 Department of Computer Science, University of Tehran, Kish International Campus, Kish, Iran; 2 Department of Mathematics, Statistics and Computer Science, University of Tehran, Tehran, Iran; Soochow University, CHINA

## Abstract

Aerobiology is a branch of biology that studies microorganisms passively transferred by the air. Bacteria, viruses, fungal spores, tiny insects, and pollen grains are samples of microorganisms. Pollen grains classification is essential in medicine, agronomy, economy, etc. It is performed traditionally (manually) and automatically. The automated approach is faster, more accurate, cost-effective, and with less human intervention than the manual method. In this paper, we introduce a Residual Cognitive Attention Network (RCANet) for the automated classification of pollen grains microscopic images. The suggested attention block, Ventral-Dorsal Ateetntion Block (VDAB), is designed based on the ventral (temporal) and dorsal (parietal) pathways of the occipital lobe. It is embedded in each Basic Block of the architecture of ResNet18. The VDAB is composed of ventral and dorsal attention blocks. The ventral and dorsal streams detect the structure and location of the pollen grain, respectively. According to the mentioned pathways, the Ventral Attention Block (VAB) extracts the channels related to the shape of the pollen grain, and the Dorsal Attention Block (DAB) is focused on its position. Three publicly pollen grains datasets including the Cretan Pollen Dataset (CPD), Pollen13K, and Pollen23E are employed for experiments. The ResNet18 and the proposed method (RCANet) are trained on the datasets and the proposed RCANet obtained higher performance metrics than the ResNet18 in the test step. It achieved weighted F1-score values of 98.69%, 97.83%, and 98.24% with CPD, Pollen13K, and Pollen23E datasets, respectively.

## Introduction

Aerobiology is the study of biological particles in the air and their diffusion mechanisms. The analysis of pollen grains plays a vital role in a wide range of applications, such as analysis of climate change, studying allergies, quality control of honey products, and crime scene evidence. Pollen grains of some plants are resistant to destruction in without oxygen environments. Therefore, this information can be used to express climate changes in those environments. Mass production of pollen grains has caused them to form part of the earth’s atmosphere. The concentration of pollen grains is estimated every year by many countries to analyze the problems related to people with allergies. Hay fever (allergic rhinitis) is a common allergy caused by many pollen grains. Therefore, identifying pollen grains is one of the most important ways to reduce sensitivity in people prone to allergies. Honey bees collect pollen from flowers and plants with their hind legs. The quality of honey produced is determined by the chemical composition of the pollen, which depends on factors such as plant origin, bee species, and geographical origin. The best method to identify the plant origin of pollen is pollinological analysis. In criminology, the release of pollen grains on the victim’s body and clothing can help identify the original location. This happens through their effect, and if the body has been moved, it can identify the type of area where the crime took place. Even if the body is buried under the soil, the grave can be identified through the growth of pollen from new and special plants on the burial site. Therefore, plant pollen can provide forensic botany with information about the location and time of the murder and the decomposition of the body [1–4].

The scientific study of pollen of plants and spores is called palynology. Palynologists identify pollens from non-pollens through a microscope and then categorize the pollen grains based on their visual features in the traditional (manual) classification. In some cases, the plant species can only be recognized by the pollen grain because the structure of the margins of its pollen grains is very special. In other cases, pollen grains with very similar structures are categorized into completely different plant species. Traditional classification of pollen grains is tedious, time-consuming, less accurate, and with full human intervention. Therefore, the automated classification of pollen grains can save time and resources [5, 6].

Handcrafted features-based and deep features-based methods are employed for automated pollen grain classification. Feature engineering (extracting and selecting discriminative features) is manually performed in the handcrafted features-based approaches. This procedure is time-consuming and dependent on the researcher’s knowledge and experience rather than deep features-based methods [7]. However, feature engineering is automatically done using deep learning, which does not have the disadvantages of the manual process. For these reasons, most researchers prefer deep learning methods for automatic pollen grain classification [8].

Various Machine Learning (ML) methods based on Deep Neural Networks (DNNs) have recently been effective for image classification [9, 10]. Convolutional Neural Networks (CNNs) are a well-known type of DNNs that has attracted the attention of researchers in the fields of Artificial Intelligence (AI) and Computer Vision (CV) in the last decade [11–13]. Each standard CNN has three layers: convolution, pooling, and fully connected. The convolution and pooling layers are at the beginning of the network, which automatically extracts the input features. Then, classification is performed by fully connected layers [14–16].

The human brain consists of about 10^11^ neurons, and the most received brain information (about 80%) are related to the visual system [17]. Therefore, it is essential in the CV field to identify more precisely how visual information processes in the brain. The human visual system is designed in such a way that it can focus on the significant information of the image or video and ignore irrelevant information. This procedure is called the attention mechanism. Bahdanau et al. proposed the first utilization of the attention mechanism in DNNs for machine translation [18]. DNNs can implement the attention mechanism in language, speech, text, vision, etc. [19, 20].

The research on the anatomy of the human visual pathways has determined that the brain’s ventral (temporal) and dorsal (parietal) pathways carry out visual perception. Identifying the architecture of the object is performed by the ventral stream, and the extraction of movement and spatial information is the responsibility of the dorsal stream [21].

In this paper, the suggested cognitive attention block is designed based on the ventral and dorsal pathways in the human brain. It is embedded in the ResNet18 architecture for automatically classifying pollen grain microscopic images. Using residual architecture can help to develop and overcome the gradient vanishing problem with increased depth of network [22].

The structure of the paper is as follows. First, in the related work section, we present previous state-of-the-art studies on the classification of pollen grains in microscopic images and the prior channel-spatial attention module. The materials and methodology section explains the used datasets, data augmentation, splitting the samples of the datasets, and the architecture of the proposed method. We describe the configuration of the training step, the performance metrics of the networks, the ablation study, the comparison performance metrics of the proposed method with the previous state-of-the-art models, the feature maps representation, and cases of correct and incorrect classification in the experiment and results section. The discussion section performs an analysis of the size of kernel (K) in the suggested attention block, the model performance metrics, feature maps, and cases of correct and incorrect classification. Finally, the conclusion and feature work sections are given.

## Related work

We explain the state-of-the-art previous related works in the field of machine learning methods and channel-spatial attention modules in this section.

### Machine learning methods

We studied and categorized previous state-of-the-art machine learning into handcrafted features-based and deep features-based methods for automated pollen grain classification and they are shown in Table 1. Studies [24, 27] generated different models by various feature extractors and classic classifiers. They compared the classification results of each approach and reported the model with better performance metrics. The models with Support Vector Machines (SVM) [23] classifiers can obtain better criteria than others because the SVM classifier is suitable for high-dimensional data and decreases the probability of overfitting. They used limited datasets for evaluating the models and obtained low accuracies. Even though the SVM is memory efficient but requires extra effort and a long training time for large datasets. The deep features-based methods are performed more than handcrafted feature-based for the pollen grains classification. CNNs have been used more among the different deep learning techniques. Transfer Learning (TL) is a method for pre-training a CNN on a large dataset and using obtained knowledge for new learning. TL and data augmentation were used with a CNN in more deep features-based methods for pollen grain classification. These approaches can improve the robustness of networks and avoid overfitting of them. Also, the features from small training sets are learned as well and with less training time by TL. However, heavily relying on large datasets to avoid overfitting is one of the important limitations of it [3]. The CNNs with these methods achieved high accuracies in studies [28, 32, 35]. All deep features-based methods (except [36]) used only one pollen grains dataset that has had low diversity for experiments and the results obtained by their models are not very good, accurate, and reliable. Three different pollen grains datasets are employed in task [36]. It has achieved superior performance in terms of accuracy and computational efficiency than all methods. The previous methods are analyzed with more details in the following.

**Table 1:** Summarization of previous related methods for automated pollen grain classification.

Study	Type	Year	Feature and Method	Used Dataset	Weighted Accuracy (%)	Weighted F1-score (%)
Goncalves et al. [24]	Human vision	2016	Human vision	Pollen23E	63.57	40.87
Goncalves et al. [24]	Handcrafted features-based	2016	Color, Shape and Texture (CST) and Bag of Words (BoW) features + K-Nearest Neighbor (K-NN), Decision Tree (DT), and Support Vector Machines (SVM) classifiers	Pollen23E	68.57	53.66
Kong et al. [25]		2016	Discriminative patch selection + Spatially Aware Coding (SACO)	Fossil pollen grain	86.13	-
Manikis et al. [26]		2019	Geometric and textural features + Random Forest (RF) classifier	Private dataset	88.24	88.07
Battiato et al. [27]		2020	Local Binary Pattern (LBP) and Histogram of Oriented Gradient (HOG) features + AdaBoost, Random Forest (RF), SVM, and Radial Basis Function (RBF) kernel SVM (RBF-SVM) classifiers	Pollen13K	86.58	85.66
Sevillano and Aznarte [28]	Deep features-based	2018	CNN (AlexNet) + Transfer Learning (TL) + Linear Discriminant Analysis (LDA) classifier	Pollen23E	97.22	96.69
Astolfi et al. [29]		2020	CNN (DenseNet-201) + Augmentation	Pollen73S	95.8	96.4
Battiato et al. [30]		2020	CNN (SmallerVGGNet) + Augmentation	Pollen13K	89.73	89.14
Sevillano et al. [31]		2020	CNN (AlexNet) features + LDA classifier + Augmentation	Private dataset	97.86	97.8
Da Silva Soares et al. [32]		2021	CNN (MobileNet) + TL + Augmentation	Pollen23E	96.5	96.75
Gui et al. [33]		2021	CNN (SmallResNet) + Augmentation	Pollen13K	97.29	97.26
Mahbod et al. [34]		2021	Ensemble CNN (EffcientNetB0, B1, B2, and SEResNeXt-50) + Augmentation	Pollen13K	96.28	96.3
Tsiknakis et al. [35]		2022	CNN + Ensemble TL + Augmentation	CPD	97.5	96.89
Mahmood et al. [36]		2023	Deep features aggregation + Squeeze and Excitation (SE) block + Augmentation	CPD Pollen23E Pollen73S	98.33 97.39 97.21	98.39 97.66 97.37

#### Handcrafted features-based methods

Battiato et al. [27] presented several methods for automatic pollen grain classification. They extracted features by Local Binary Pattern (LBP) [37] and Histogram of Oriented Gradient (HOG) [38]. The classification is performed by classic classifiers including AdaBoost [39], Random Forest (RF) [40], Linear SVM, Radial Basis Function (RBF) kernel SVM (RBF-SVM) [41]. They used the Pollen13K dataset [30] for experiments. 85% of the dataset was divided as the training set and 15% as the test set. The methods were trained and evaluated with both types of feature extraction, and RBF-SVM using the HOG features obtained the highest values in terms of weighted accuracy with 86.58% and weighted F1-score with 85.66% in the test step. Although they used many classic machine-learning methods, the methods were implemented on one dataset with limited pollen grain classes, and their methods obtained low-performance metrics.

Gonçalves et al. [24] proposed several supervised machine-learning methods for pollen grain classification. They used a combined Color, Shape, Texture (CST), and Bag of Words (BoW) for feature extraction and SVM, Decision Tree (DT) [42], and K-Nearest Neighbor (K-NN) [43] for classification. The dataset was also evaluated by human vision. The methods implemented on the Pollen23E dataset [28] and SVM obtained better performance metrics than others with a weighted accuracy and weighted F1-score of 68.57% and 53.66% on the test step, respectively. The human vision method classified the samples of the dataset with 63.57% weighted accuracy and 40.87% weighted F1-score in the test step. Two methods achieved very low-performance metrics in his study.

#### Deep features-based methods

Mahmood et al. [36] proposed an attention-guided pollen feature aggregation network (APFA-Net) for classifying pollen grains. The backbone of APFA-Net is composed of four convolution groups. Each of them has four feature aggregation nodes [44] and two modified squeeze-and-excitation (SE) blocks [45]. They used three datasets, Certain Pollen Dataset (CPD) [46], Pollen23E, and Pollen73S [29] in their experiments. Data augmentation techniques are applied in the training set of the datasets. The method was trained on them with the same conditions. It achieved weighted accuracies of 98.33%, 97.39%, and 97.21% and weighted F1-scores of 98.39%, 97.66%, and 97.37% with CPD, Pollen23E, and Pollen73S datasets in the test step, respectively. Authors focus on channel-wise information by modifying the SE block but pay less attention to the border of the pollen grain or the Region of Interest (RoI) of feature maps. When the method is focused on the feature related to the border of the pollen grain and is ignored irrelevant features can extract important features. Therefore, the classification is improved by features related to the shape of pollen grains.

Tsiknakis et al. [35] investigated a method by combining transfer and ensemble learning [47, 48] for the classification of pollen grain images. They employed augmentation techniques to increase the size of the training set. The method was trained and evaluated on the CPD dataset. It attained a weighted accuracy and weighted F1-score of 97.5% and 96.89% in the test step, respectively. Although they used state-of-the-art deep learning methods in this article, the method has been examined only on a dataset. Also, it has not achieved very high-performance metrics.

Da Silva Soares et al. [32] applied MobileNet [49] and transfer learning to classify pollen grains images. They performed two experiments (with and without data augmentation) with the Pollen23E dataset. The method achieved 92% weighted accuracy in the worst case and 100% weighted accuracy in the best case in two states. It is surprising to obtain similar accuracies in the best and worst case of two different types of experiments. Although the method has been implemented with four different experiments on the dataset, it would have been better if these states were performed on other pollen grain datasets to obtain a more accurate evaluation of the method.

Gui et al. [33] presented a CNN method with a ResNet backbone using Mask Complement and Cut Occlusion operations to classify pollen grains images automatically. The method had three steps. In the first step, the Mask Complement was modified by dilation operation to complete the missing areas of the original images. In the second step, the Cut Occlusion operation created patches of the center point to the edges of the image and made it into a black occlusion during the training process. Samples from prior steps were employed in the third step to train the method and to predict the pollen grains class in the test phase. Other data augmentation methods such as salt and pepper noise [50], geometric and color transformation [51] were used to increase the number of the Pollen13K dataset images. They separated 80% of the Pollen13K dataset for the train and 20% for the test step. The method was trained and achieved weighted accuracy and weighted F1-score of 97.29% and 97.26%, respectively, in the evaluation phase. They performed experimental evaluations on a limited dataset.

Mahbod et al. [34] proposed a CNN-based fine-tuned ensemble method for automatic pollen grains classification. The method consisted of four steps: pre-processing, pre-trained, fine-tuning, and fusion. Mean intensity RGB values of the ImageNet dataset [52] were reduced for training and test images in the pre-processing, and the photos were resized to 260 × 260 pixels. EffcientNet and SEResNeXt [45] were employed for pre-training. Therefore, they used four sub-networks including three EffcientNetB0, three EffcientNetB1, three EffcientNetB2, and three SeResNeXt-50 in the backbone of the CNN method. The CNN was fine-tuned with three different image sizes (224 × 224, 240 × 240, and 260 × 260 pixels). The Ensemble technique was used for classification. Each sub-network has fused the results of five folds in each image size. Therefore, the results of three different image sizes in each sub-network were combined, and the maximum probability of sub-networks was fused as the final prediction vector. In other words, the final prediction vector was determined by taking the average over the prediction vectors of the sub-networks. The network was implemented on the Pollen13K dataset and achieved weighted accuracy and weighted F1-score of 96.28% and 96.3%, respectively, in the test phase. They used simple techniques in the architecture of the method and they also evaluated their method only on a pollen grain dataset.

Battiato et al. [30] proposed two CNN methods, AlexNet and SmallerVGGNet (Very Deep Convolutional Networks (VGGNet)) [53], too. They trained and tested the methods with and without data augmentation on the Pollen13K dataset for 30 epochs and measured the weighted accuracy and weighted F1-scores of the models in both train and test steps every ten epochs. The SmallerVGGNet with data augmentation obtained the highest values with a weighted accuracy of 89.73% and a weighted F1-score of 89.14% in the test step. The used methods are simple and have obtained low classification results.

Sevillano and Aznarte [28] suggested a state-of-the-art deep learning method that is composed of pre-trained AlexNet with transfer learning and Linear Discriminant Analysis (LDA) [54] for pollen grains classification. The method was implemented on the Pollen23E and it achieved a weighted accuracy of 97.22% and a weighted F1-score of 96.69% in the test step. Using one dataset for evaluating the method and obtaining low-performance metrics are weaknesses of this study.

### Channel-spatial attention modules

Attention modules are employed in the image classification tasks to make a network for learning and focusing more on the RoI in the feature maps. They improve classification results by extracting useful information related to the RoI. A channel-spatial module is composed of the benefits of channel attention and spatial attention that choose the important objects and regions of the feature maps. There are two state-of-the-art channel-spatial attention modules, the Convolutional Block Attention Module (CBAM) and the Bottleneck Attention Module (BAM), which are described in the following.

#### Convolutional Block Attention Module (CBAM)

The first channel-attention module called CBAM that introduced by Woo et al. [55]. It has two sub-modules including the channel and spatial attention modules which apply channel and spatial relations of features. The CBAM could be placed inside residual blocks as well as at the bottleneck.

#### Bottleneck Attention Module (BAM)

Park et al. [56] proposed the second channel-attention module named BAM. It has two sub-modules including the channel and spatial attention modules which utilize channel and spatial relationships of features. The BAM has a bottleneck architecture to save computational costs by using a dilated convolution in the spatial attention sub-module structure. Dilated convolution increases the receptive field and produces a bottleneck for the downsampling of feature maps. The BAM could employed at each bottleneck of the architecture of ResNet.

**Fig 1:**
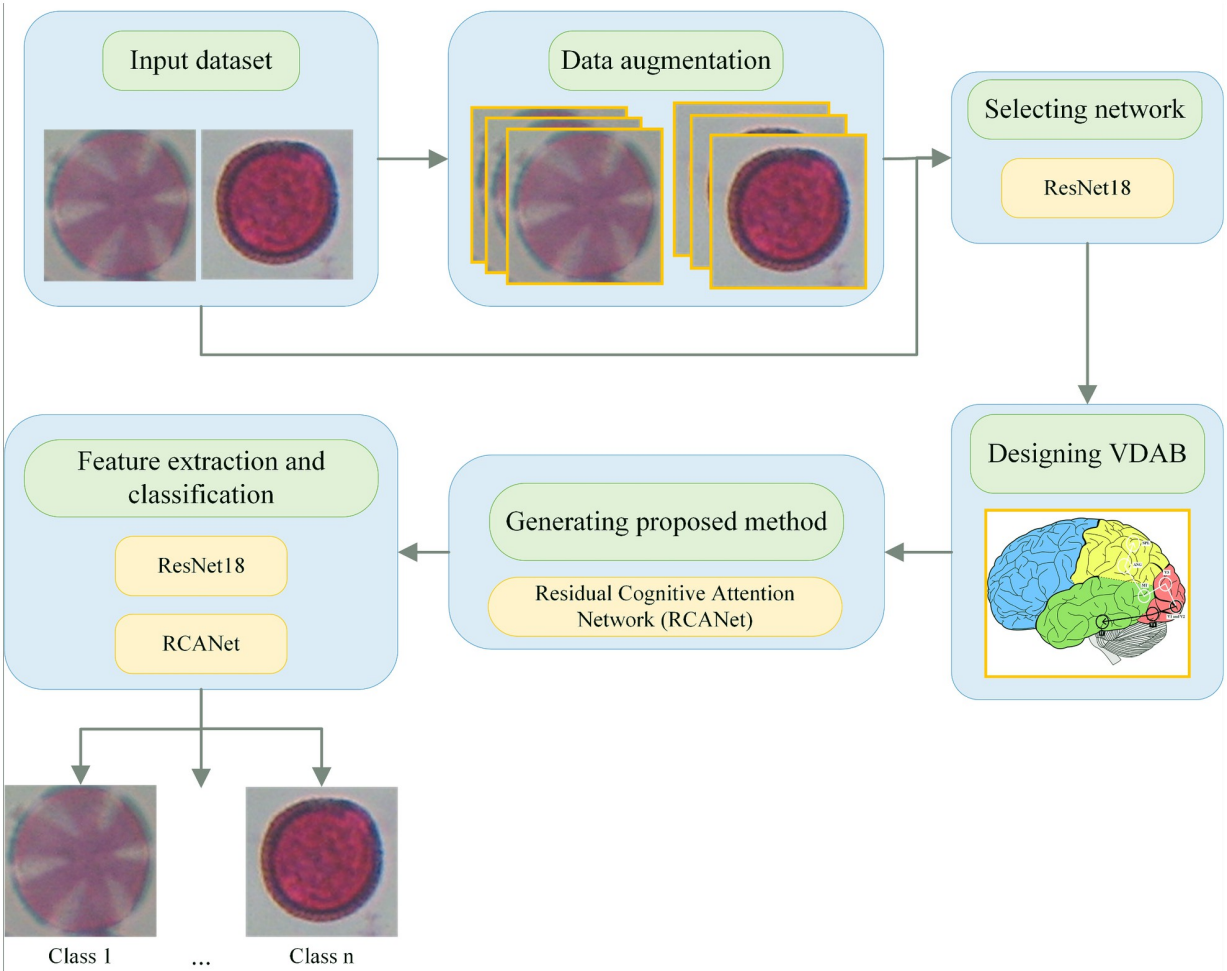
The graphical representation of the overall proposed scheme.

The graphical representation of the overall proposed scheme in this paper is displayed in Fig 1.

## Materials and methodology

We describe the used datasets and the architecture of the proposed method in this section.

### Used datasets

We used three state-of-the-art pollen grain datasets including CPD, Pollen13K, and Pollen23E. The reasons for choosing these datasets in this study are as follows:

- They have been more employed in recent years.
- The datasets were different in the number of classes of pollen grains.
- The classes in the Pollen23E dataset were balanced while the classes in the CPD and Pollen13K datasets were unbalanced.
- The number of their training samples was suitable for the proposed method that avoids underfitting and overfitting them during training.
- The datasets have different origins in terms of geographic location and type.

Therefore, the proposed method will be trained and evaluated more comprehensively with selected datasets and the results will be more accurate and reliable. The details of each of the used datasets are explained in the following.

#### CPD

**Fig 2:**
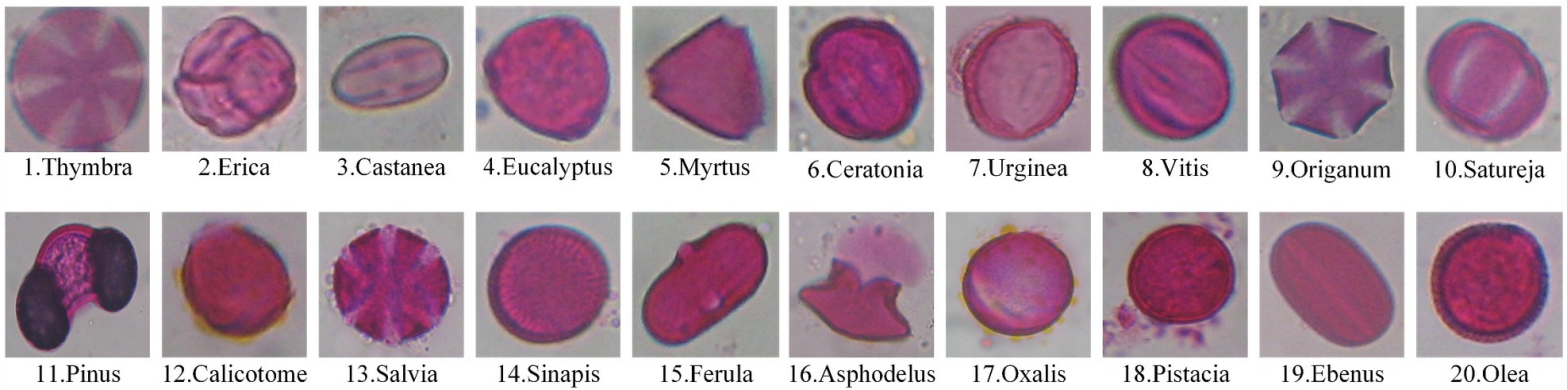
Sample images of each class of the CPD.

Tsiknakis et al. introduced the CPD in 2021. It contains 4034 pollen grain microscopic images from 20 different categories which were collected from different places in the region of Crete, Greece from April 2019 to April 2021. The origin images of the CPD dataset are plants and have different resolutions [46]. Sample images of each class of the CPD are shown in Fig 2.

**Fig 3:**
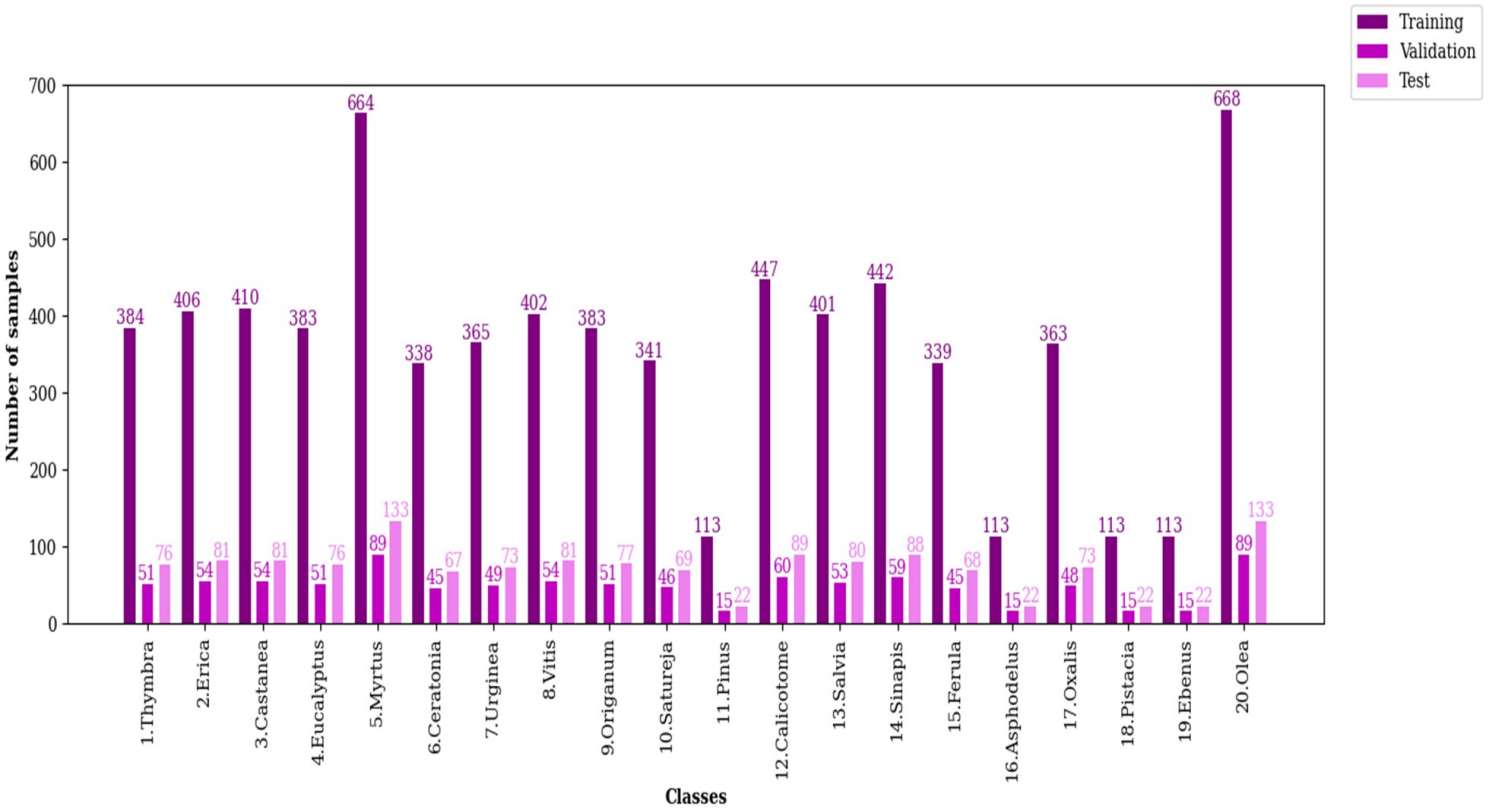
The histogram of each class of the CPD.

One of the reasons for model overfitting is that there is not enough data in the training set. Using different data augmentation techniques increases the data and avoids overfitting [57–59]. Since the number of samples of the CPD is small, we employed different data augmentation methods such as brightness, flipping (horizontal and vertical), horizontal shift (left and right), rotation, scaling, shearing, and vertical shift (down and up) for increasing its data. The histogram of each class of the training, validation, and test sets of the CPD after data augmentation is shown in Fig 3.

#### Pollen13K dataset

**Fig 4:**
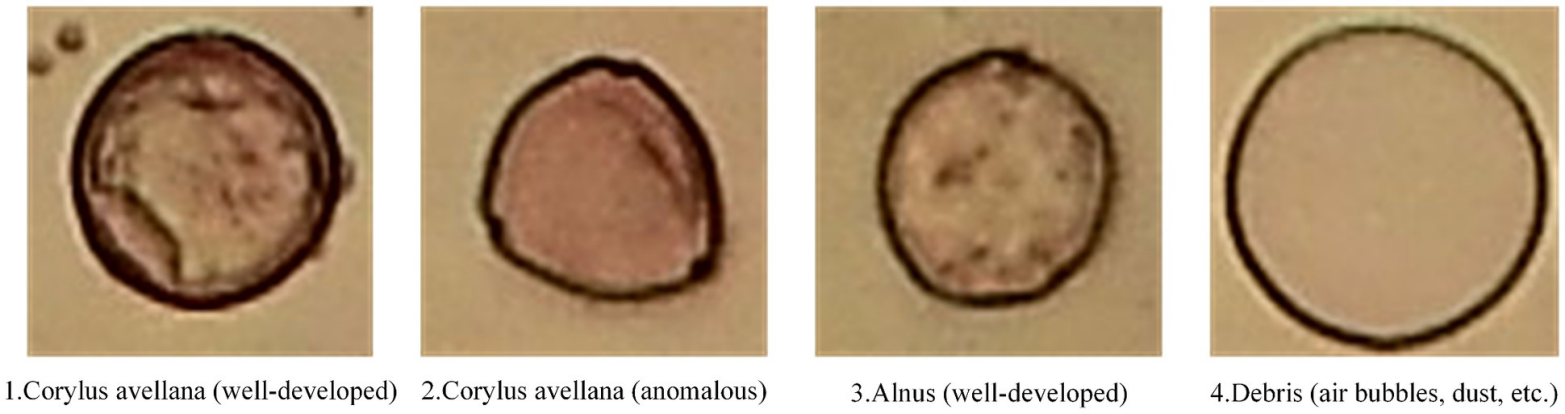
Sample images of each class of the Pollen13K dataset.

**Fig 5:**
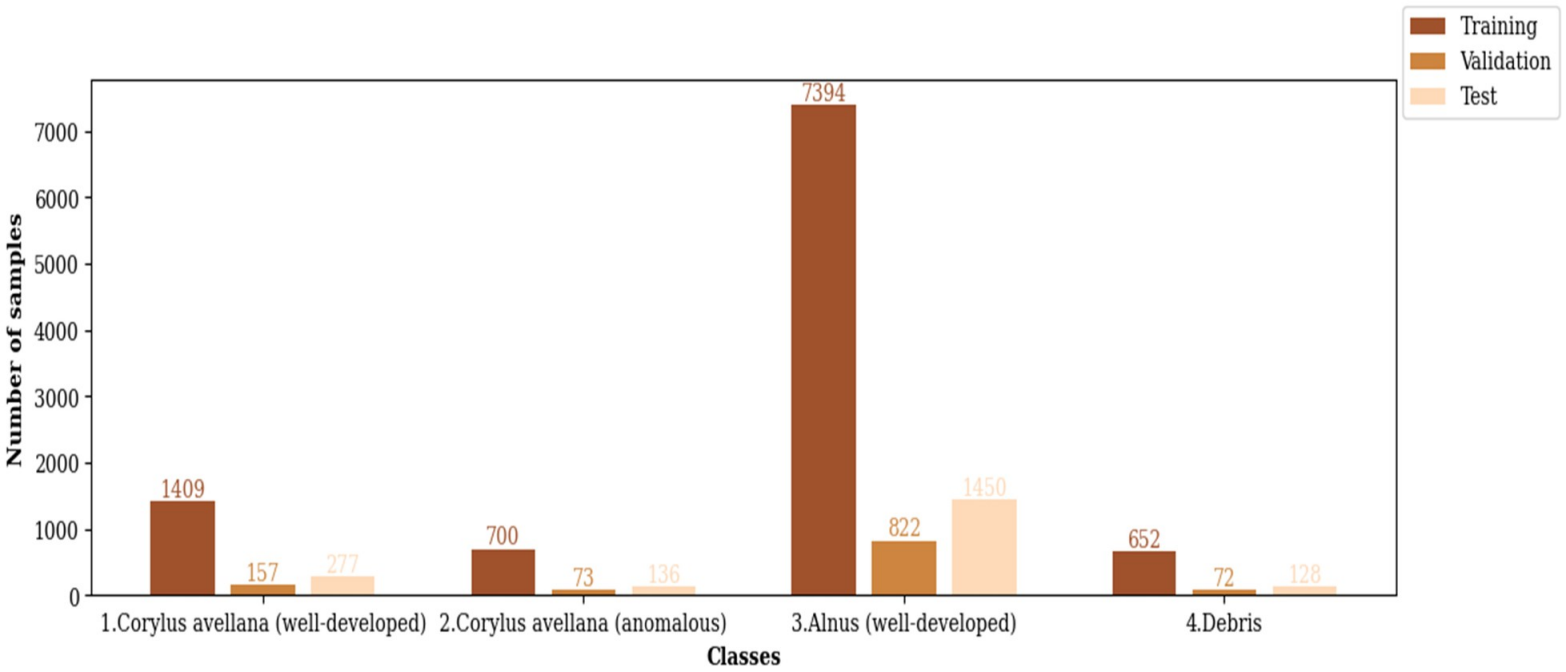
The histogram of each class of the Pollen13K dataset.

Battiato et al. proposed the Pollen13K dataset in 2020. It contains 13,270 pollen grain microscopic images that are classified into four different classes [30]. The origin samples of the Pollen13K are airborne and have 84 × 84 resolution [46]. Sample images of each class and data histogram of each class of the training, validation, and test sets of the Pollen13K dataset are shown in Figs 4 and 5, respectively.

#### Pollen23E dataset

**Fig 6:**
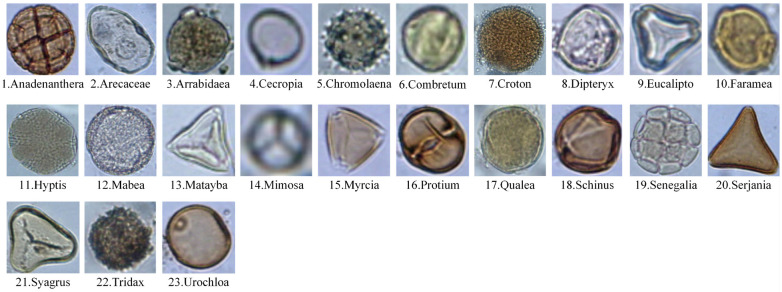
Sample images of each class of the Pollen23E dataset.

**Fig 7:**
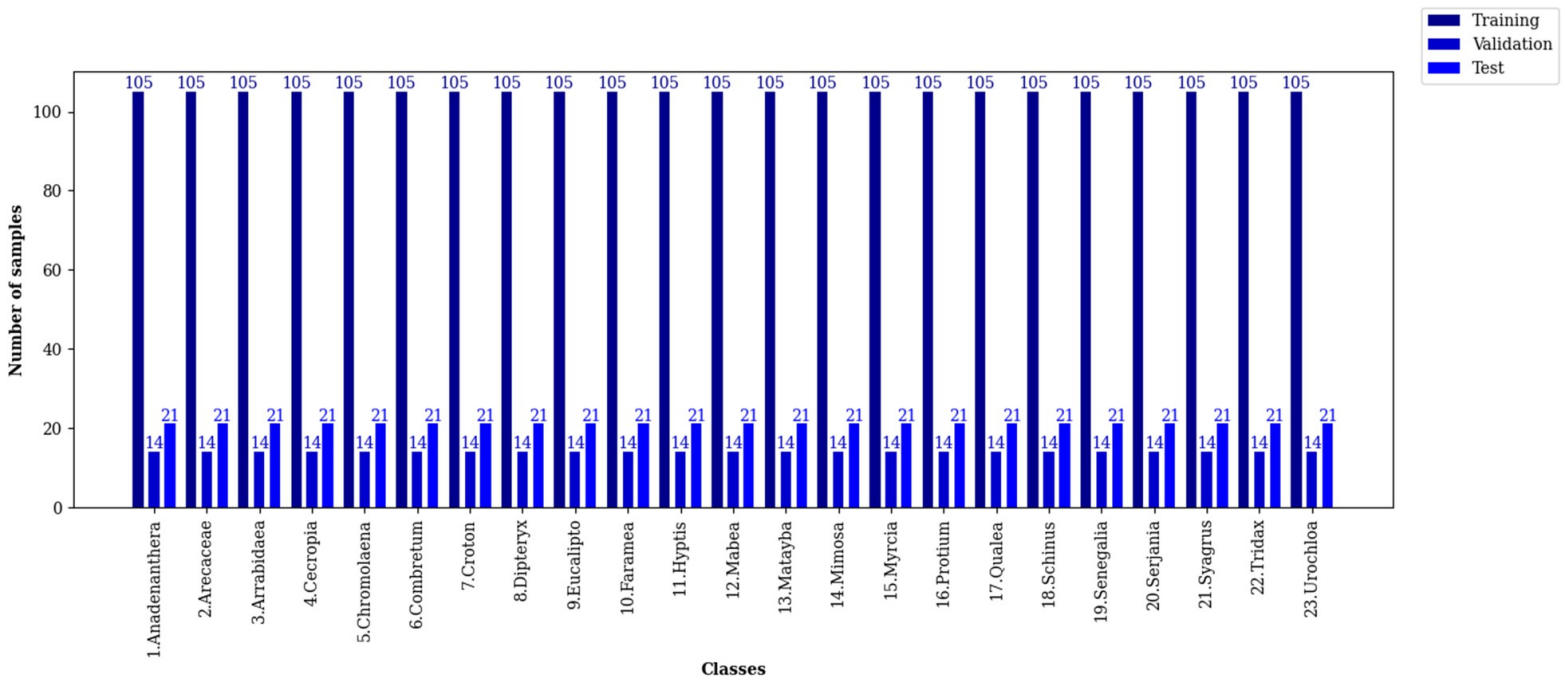
The histogram of each class of the Pollen23E dataset.

Gonçalves et al. suggested the Pollen23E dataset in 2016. It comprises 805 pollen grain microscopic images from 23 different types that are present in the Brazilian Savannah [24]. The origin images of the Pollen23E dataset are honey and have a variety of resolutions [46]. Sample images of each class of the Pollen23E dataset are shown in Fig 6. Due to the lack of training data on the Pollen23E dataset, we employed data augmentation methods (like data augmentation in the CPD) to enrich it. The histogram of each class of the training, validation, and test sets of the Pollen23E dataset after data augmentation is shown in Fig 7.

### Splitting the samples of the datasets

We separated the samples of each dataset into two different kinds including the train-validation-test and K-fold cross-validation splits for improving the effectiveness, robustness, and generalization ability of the networks, as well as avoiding underfitting and overfitting. The used train-validation-test split and K-fold cross-validation in this study are explained in the following.

#### Train-validation-test split

One of the most common and critical techniques for assessing a predictive model is to divide data into training, validation, and test sets. The training set is used to fit the model and tuning the model’s parameters is performed in the validation step. Unseen data in the test set are employed for the model’s final performance. Therefore, the train-validation-test split helps evaluate how well a model will generalize to the test data and also prevent overfitting. For the first set of experiments, each dataset is divided into training, validation, and test sets based on Table 2. These sets contain 75%, 10%, and 15% of the dataset images, respectively.

**Table 2:** Splitting train-validation-test of data.

Dataset	Training Set	Validation Set	Test Set	Total Data
CPD	7,188	958	1,433	9,579
Pollen13K	10,155	1,124	1,991	13,270
Pollen23E	2,415	322	483	3,220

#### K-fold cross-validation split

**Fig 8:**
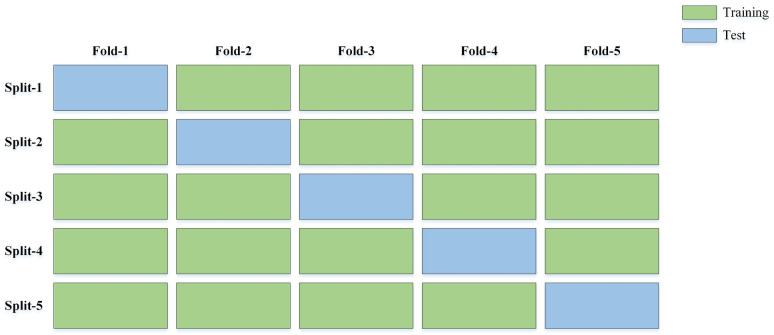
Diagram of the 5-fold cross-validation procedure.

The k-fold Cross-validation technique is used to improve the effectiveness, robustness, and generalization ability of deep learning models, as well as to avoid overfitting. For the second set of experiments, we first separated every dataset into the training and test sets, comprising 90% and 10% of the samples respectively based on Table 3. Then, the 5-fold cross-validation procedure is employed according to Fig 8.

**Table 3:** Splitting of data for 5-fold cross-validation.

Dataset	Training Samples	Test Samples	Total Data
CPD	8,146	1,433	9,579
Pollen13K	11,279	1,991	13,270
Pollen23E	2,737	483	3,220

### Two Visual Streams Hypothesis (TVSH)

The capacity of the brain is limited but it receives massive of visual information. Therefore, it pays attention only to the essential information. Cognitive attention is the visual procedure that selects relevant information and filters out irrelevant information. This selection focuses more on particular regions of space, specific features of an object, or entire of them in a real or imagined scene [60]. According to the Two Visual Streams Hypothesis (TVSH), the visual information in the brain first enters the Visual area one (V1) and Visual area two (V2) of the occipital lobe. Then, they are sent to the ventral and dorsal pathways. The ventral stream continues from V2 to the Visual area fourth (V4) and Inferior Temporal (IT). It extracts the characters of the structure of the objects or the “what-information” processing area. The dorsal pathway continues from V2 to the Visual area three (V3), the Visual area fifth (V5) or Medial Temporal visual area (MT), the Angular gyrus area (ANG), and the Superior Parietal Lobule (SPL). It identifies spatial organization, guiding action, and spatial attention or the “where-information” processing area [21]. We design cognitive attention blocks inspired by TVSH and the CBAM in the architecture of the proposed method.

### Architecture of the proposed method

**Fig 9:**
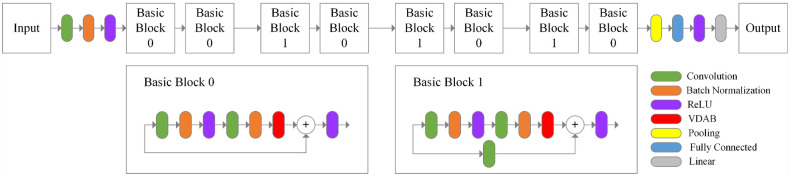
The architecture of the proposed RCANet.

The architecture of the proposed method, Residual Cognitive Attention Network (RCANet), for automatically classifying microscopic images of pollen grains is shown in Fig 9. ResNet18 is the backbone of the proposed RCANet. We fine-tuned ResNet18 before doing experiments on the datasets. ResNet18 is designed for 1000 classes and we modified it based on the classes of each of the datasets. The main components of the structure of ResNet18 are Basic blocks. We used the suggested cognitive attention blocks within the Basic Blocks of the proposed RCANet.

In the following, each component of the proposed RCANet is described in detail.

#### Residual blocks

**Fig 10:**
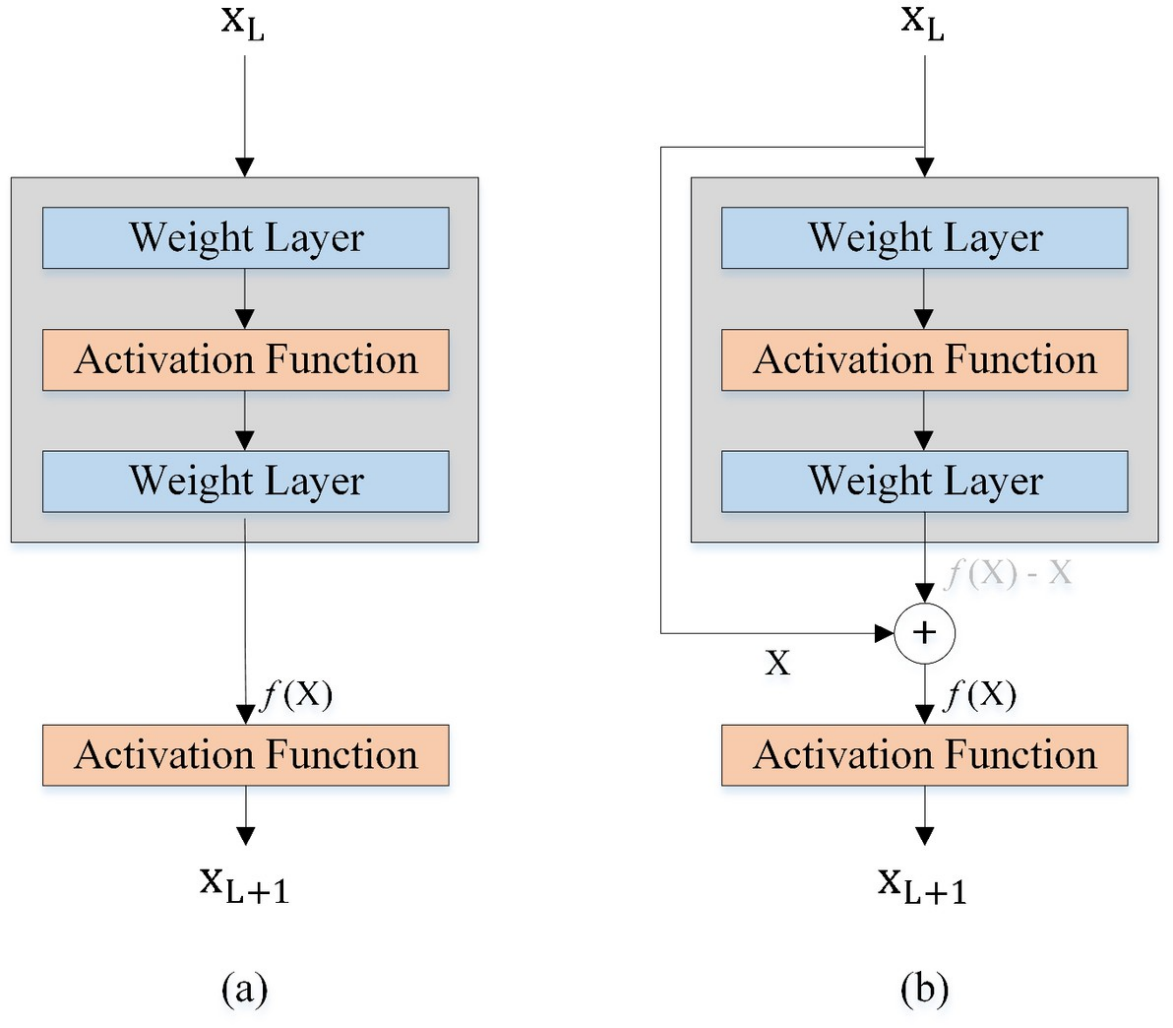
Regular and residual blocks. (a) regular block, (b) residual block.

Before introducing the concept of residual blocks in ResNet, there were challenges of the vanishing or exploding gradient, model overfitting, and higher learning error with the increase in the number of layers in DNNs [22]. The structure of a regular block and a residual block is illustrated in Fig 10. We assume X_L_ is the input of the L layer and X_L+1_ is the output of the L layer (the input of the next layer (L+1)), and what we want to achieve by learning is called *f*(x) or the desired underlying mapping. The area within the grey box must directly learn the mapping *f*(x) in the regular block. But in the residual block, it needs to be learned by the residual mapping *f*(x)-x. The solid line transferring the layer input x to the addition operation is named a residual (shortcut) connection. The output of previous layers can be faster propagated as input of the current layer through the residual connection path. If we set the weights and biases of the lower weight layer to zero, the grey box in the residual block is equal to zero (*f*(x)=x), and then the residual mapping is easier to learn.

#### Basic Blocks

**Fig 11:**
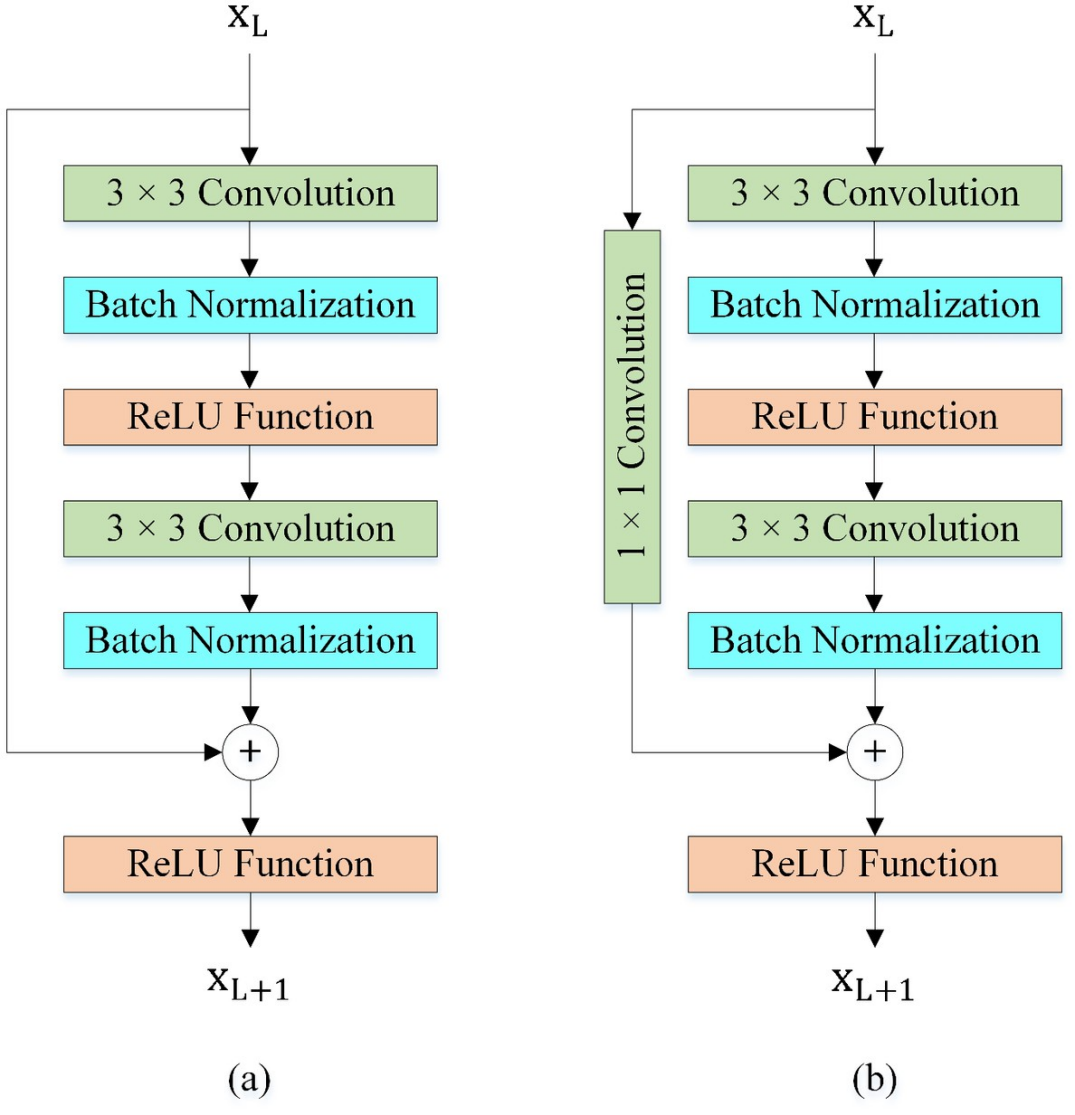
Basic Blocks. (a) Basic Block 0, (b) Basic Block 1.

Fig 11 shows the Basic Blocks in the proposed method. Both Basic Blocks are composed of 3 × 3 convolution layers, Batch Normalization (BN) layers [61], and the Rectified Linear Unit (ReLU) activation functions [62]. Basic Block 0 is used when inputs of addition operation have the same shapes (channels). Basic Block 1 can convert the input into the desired shape for the addition operation by 1 × 1 convolution layer.

#### Ventral-Dorsal Attention Block (VDAB)

**Fig 12:**
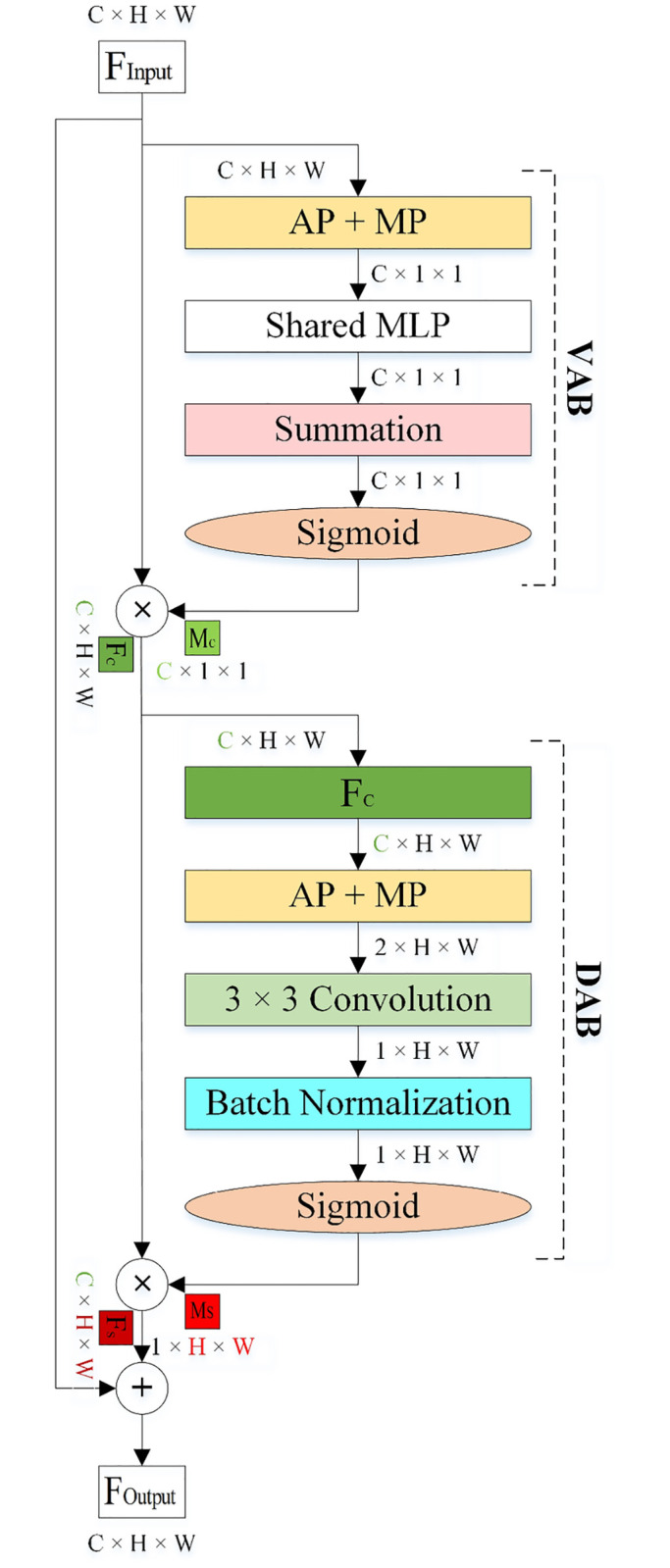
The architecture of the VDAB.

The architecture of the VDAB is shown in Fig 12. Since the VDAB is inspired by the visual perception of the human brain, It is composed of Ventral Attention Block (VAB) and Dorsal Attention Block (DAB). They are sequentially connected based on the TVSH hypothesis and the VAB is designed before the DAB. The VAB enhances and extracts feature maps that contain information related to the structure of the pollen grain while the DAB focuses on the location of the pollen grains in the selected feature maps. Therefore, by modifying the weights of the channels related to the shape and position of the pollen grain in the feature maps, the VDAB forces the proposed RCANet to pay attention to which feature maps (what-information) and where of them (where-information). As a result, the proposed RCANet can improve decision-making by extracting effective feature maps related to the structure and location of pollen grains. We explain the function of the VAB and the DAB mathematically in the following section.

**Fig 13:**
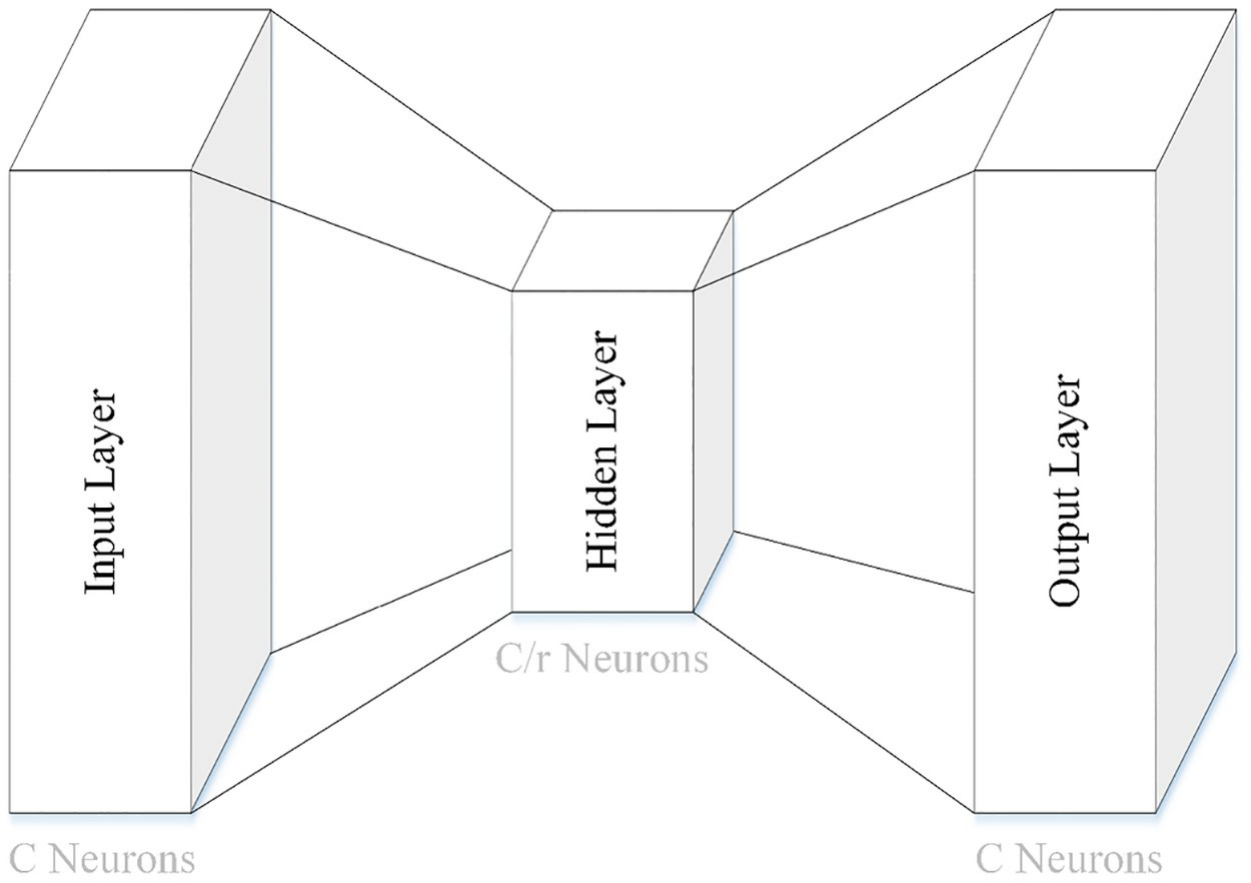
The architecture of the shared MLP.

The steps of the VAB in the VDAB are as follows:

- Average Pooling (AP) and Max Pooling (MP) are applied to the input feature map $F_{\text{Input}}\in R^{C\times H\times W}$ and two 1-Dimension feature maps including average-pooled features (${\text{F}}_{\text{AP}}^{{\text{c}}_{\text{VDAB}}}\in R^{C\times 1\times 1}$) and max-pooled features (${\text{F}}_{\text{MP}}^{{\text{c}}_{\text{VDAB}}}\in R^{C\times 1\times 1}$) are generated. **F**_**Input**_ is the input image, where *C* is the number of Channels and *H* and *W* are the Height and Width of the input, respectively.
-
${\text{F}}_{\text{AP}}^{{\text{c}}_{\text{VDAB}}}$ and $F_{\text{MP}}^{{\text{c}}_{\text{VDAB}}}$ are forwarded to a small shared Multi-Layer Perceptron (MLP) with one hidden layer for producing the other two pooled features (${F^{\prime }}_{\text{AP}}^{{\text{c}}_{\text{VDAB}}}\in R^{C\times 1\times 1}$ and ${F^{\prime }}_{\text{MP}}^{{\text{c}}_{\text{VDAB}}}\in R^{C\times 1\times 1}$). The shared MLP has two weights (${\text{W}}_{0}\in R^{C/r\times C}$ and ${\text{W}}_{1}\in R^{C\times C/r}$). The weights are shared for $F_{\text{AP}}^{{\text{c}}_{\text{VDAB}}}$ and $F_{\text{MP}}^{{\text{c}}_{\text{VDAB}}}$. The number of neurons in the hidden layer can be manually set and control parameter overhead in the shared MLP by *C*/*r*, where *r* is the reduction ratio (r). The ReLU activation function is employed in the shared MLP. The architecture of the shared MLP in the VAB of the VDAB is demonstrated in Fig 13.
-
${F^{\prime }}_{\text{AP}}^{{\text{c}}_{\text{VDAB}}}$ and ${F^{\prime }}_{\text{MP}}^{{\text{c}}_{\text{VDAB}}}$ are merged using element-wise summation (⊕).
- Sigmoid function (*σ*) is applied, and the channel attention Map is generated ${\text{M}}_{{\text{c}}_{\text{VDAB}}}\in R^{C\times 1\times 1}$. In short, ${\text{M}}_{{\text{c}}_{\text{VDAB}}}$ is calculated as:
  $$\begin{array}{c} {\text{M}}_{{\text{c}}_{\text{VDAB}}}=\sigma \left(MLP\left(AP\left(F_{\text{Input}}\right)\right)⊕MLP\left(MP\left(F_{\text{Input}}\right)\right)\right) \end{array}$$ (1)
-
${\text{M}}_{{\text{c}}_{\text{VDAB}}}$ is multiplied with **F**_**Input**_ using element-wise multiplication (⊗), and the output of the channel attention module is produced $F_{{\text{c}}_{\text{VDAB}}}\in R^{C\times H\times W}$. In summary, $F_{{\text{c}}_{\text{VDAB}}}$ is computed as:
  $$\begin{array}{c} F_{{\text{c}}_{\text{VDAB}}}={\text{M}}_{{\text{c}}_{\text{VDAB}}}⊗F_{\text{Input}} \end{array}$$ (2)

The steps of the DAB in the VDAB are as follows:

- AP and MP are applied to the $F_{{\text{c}}_{\text{VDAB}}}$ and two 2-Dimension feature maps, including average-pooled features ($F_{\text{AP}}^{{\text{s}}_{\text{VDAB}}}\in R^{1\times H\times W}$) and max-pooled features ($F_{\text{MP}}^{{\text{s}}_{\text{VDAB}}}\in R^{1\times H\times W}$) are generated. Then, $F_{\text{AP}}^{{\text{s}}_{\text{VDAB}}}$ and $F_{\text{MP}}^{{\text{s}}_{\text{VDAB}}}$ are concatenated and $F_{\text{AP},\text{MP}}^{{\text{s}}_{\text{VDAB}}}\in R^{2\times H\times W}$ is produced.
-
$F_{\text{AP},\text{MP}}^{{\text{s}}_{\text{VDAB}}}$ is forwarded to a 3 × 3 convolution layer for generating $F_{{\text{Conv}}_{3\times 3}}^{\text{s}}\in R^{1\times H\times W}$.
-
$F_{{\text{Conv}}_{3\times 3}}^{\text{s}}$ is passed to a BN layer for scale adjustment.
- Sigmoid function is applied, and the spatial attention Map is produced ${\text{M}}_{{\text{s}}_{\text{VDAB}}}\in R^{1\times H\times W}$. In short, ${\text{M}}_{{\text{s}}_{\text{VDAB}}}$ is computed as:
  $$\begin{array}{c} {\text{M}}_{{\text{s}}_{\text{VDAB}}}=\sigma \left(BN\left(Conv^{3\times 3}\left(\left[AP\left(F_{\text{Input}}\right);MP\left(F_{\text{Input}}\right)\right]\right)\right)\right) \end{array}$$ (3)
-
${\text{M}}_{{\text{s}}_{\text{VDAB}}}$ is multiplied by the $F_{{\text{c}}_{\text{VDAB}}}$ using element-wise multiplication, and the output of the DAB is generated $F_{{\text{s}}_{\text{VDAB}}}\in R^{C\times H\times W}$. In summary, $F_{{\text{s}}_{\text{VDAB}}}$ is computed as:
  $$\begin{array}{c} F_{{\text{s}}_{\text{VDAB}}}={\text{M}}_{{\text{s}}_{\text{VDAB}}}⊗F_{{\text{c}}_{\text{VDAB}}} \end{array}$$ (4)
- Finally, **F**_**Input**_ is added by $F_{{\text{s}}_{\text{VDAB}}}$ using element-wise summation and the final feature map of the VDAB is produced $F_{\text{Output}}^{\text{VDAB}}\in R^{C\times H\times W}$. In short, $F_{\text{Output}}^{\text{VDAB}}$ is calculated as:
  $$\begin{array}{c} F_{\text{Output}}^{\text{VDAB}}=F_{\text{Input}}⊕F_{{\text{s}}_{\text{VDAB}}} \end{array}$$ (5)

#### Basic Blocks with VDAB

**Fig 14:**
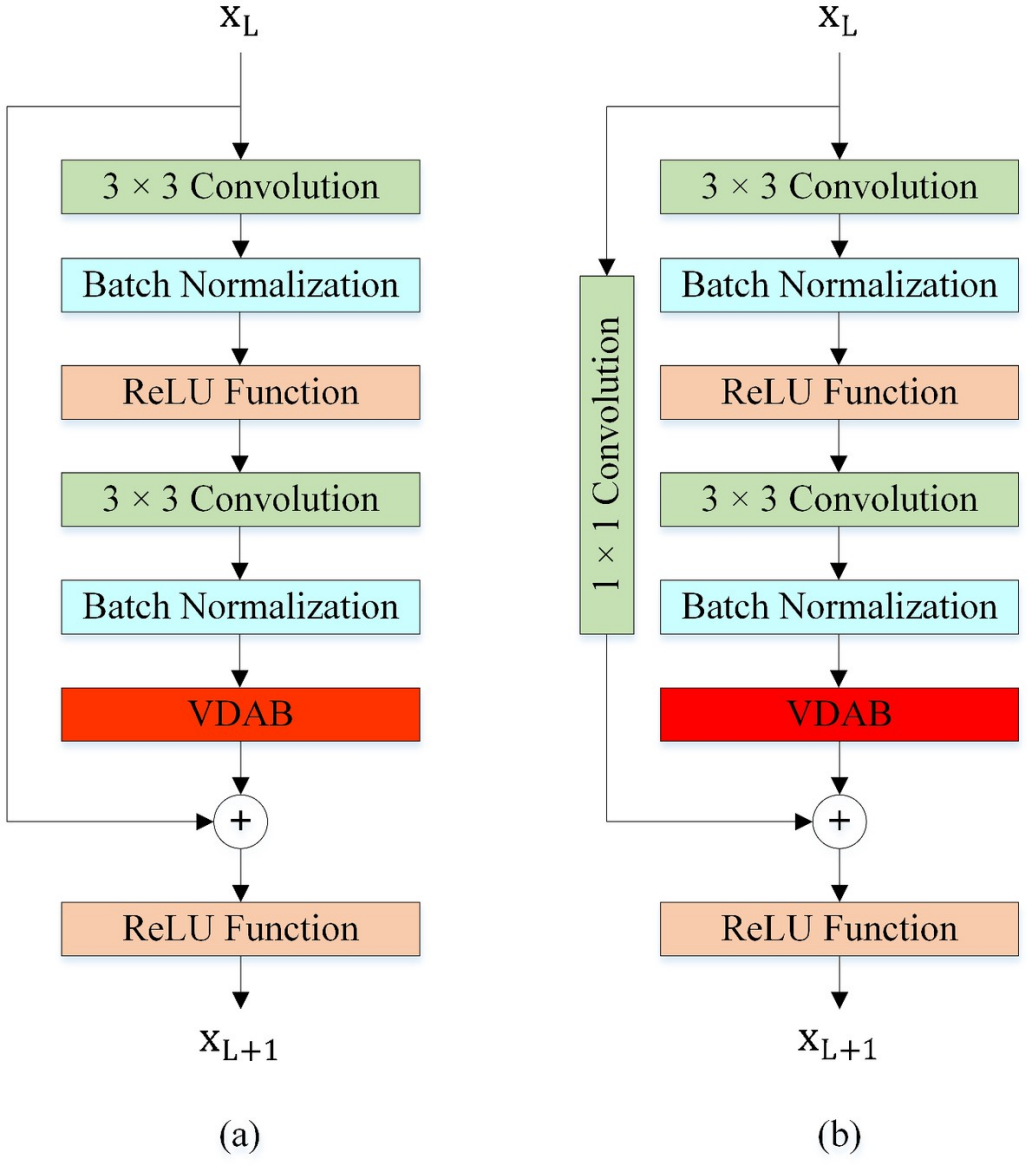
The Basic Blocks with VDAB. (a) Basic Block 0 with VDAB, (b) Basic Block 1 with VDAB.

We embedded and evaluated the different positions of the VDAB in the Basic Blocks. The test results showed that utilizing the VDAB after the second BN layer is more efficient. The location of the VDAB in the Basic Blocks is shown in Fig 14.

## Experiment and results

The configuration of the training and evaluation steps, performance metrics of the networks, ablation study, comparison with previous state-of-the-art methods, feature maps representation, and the cases of correct and incorrect prediction are explained in this section.

### The training step

**Fig 15:**
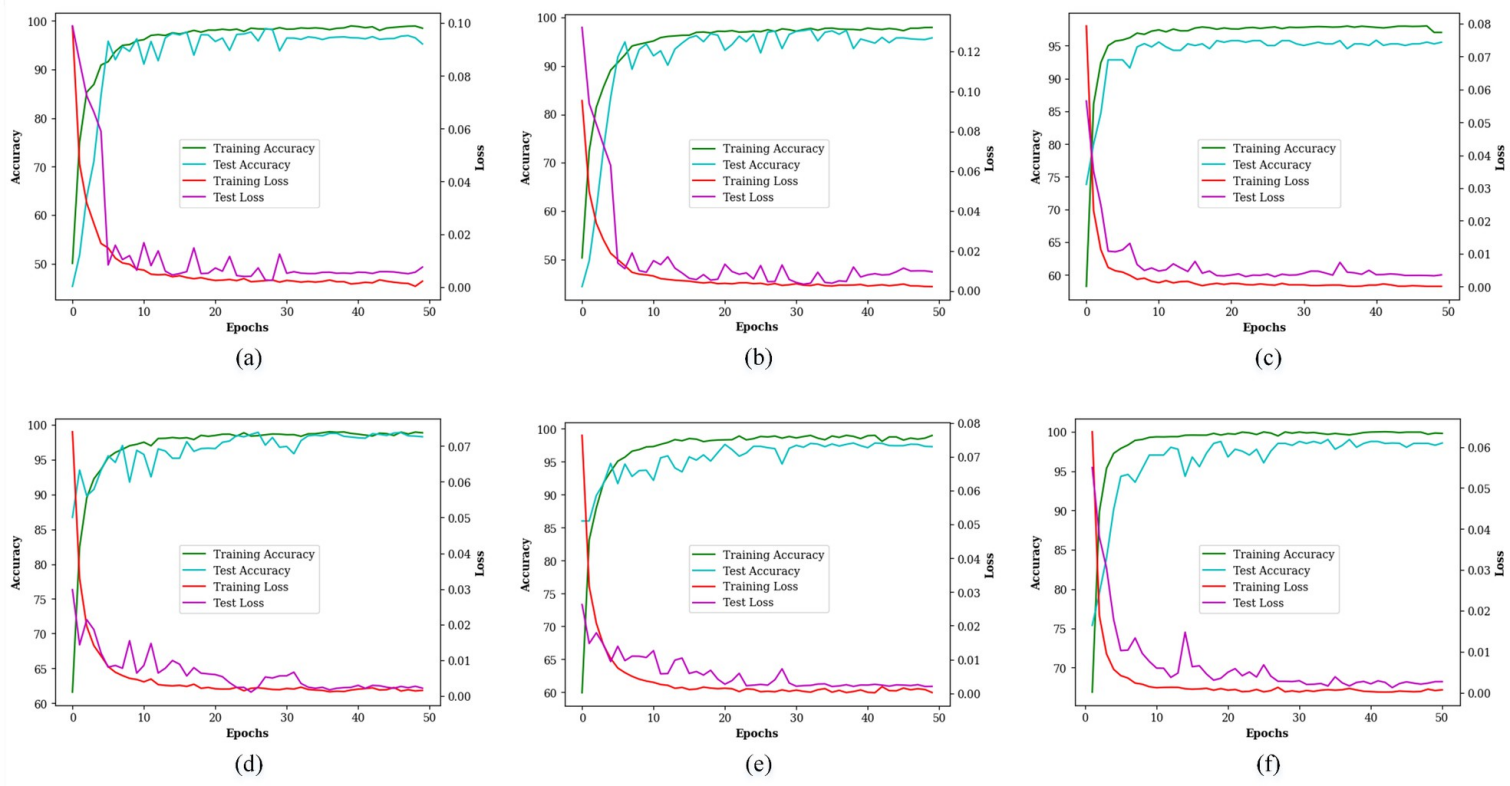
The accuracy and loss curves of the ResNet18 and the proposed RCANet during the training and test steps on the datasets. (a, d) CPD, (b, e) Pollen13k, and (c, f) Pollen23E.

In this study, we applied a workstation based on Windows 11 with a 64-bit operating system, an Intel(R) Core (TM) i7-8565U CPU, and an Intel (R) UHD Graphics 620 GPU. Python 3.10.12 and Pytorch 2.1.0 were the development environments, and the codes were run with a Graphics Processing Unit (GPU) on the internet for faster training of the networks. The images of the Pollen13K dataset have 84 × 84 pixels while the images of the CPD and the Pollen23E datasets have different sizes. Thus, the samples of the CPD and the Pollen23E datasets are resized to 84 × 84 pixels before transferring them to the network. Hyperparameters are configuration variables that are set before the training step to achieve the best of them. They have a significant impact on the performance of the network in the test step and its generalization, reducing overfitting and training time. Configurations of hyperparameters for the training step of the ResNet18 and the proposed RCANet are displayed in Table 4. The optimal hyperparameter values of the networks were experimentally determined on the training set which presented the best classification accuracy with them. We also used the Cross-Entropy loss function, the Stochastic Gradient Descent (SGD) optimizer, and a Momentum of 0.9 for our experiments. The number of filters in both networks gradually increased to learn more complex features of the training sets. The ResNet18 and the proposed RCANet are trained and evaluated with the same condition for determining the effect of the VDAB on the performance metrics of the proposed method. Fig 15 illustrates the accuracy and loss curves of the ResNet18 (a, b, and c) and the proposed RCANet (d, e, and f) during the training and test steps on the datasets. It confirms the training and test processes were successfully converged and the networks have not overfitted.

**Table 4:** Configurations of hyperparameters for the training of the ResNet18 and the proposed RCANet.

Hyperparameter	Variables	Selected
Batch size	4, 8, 16, 32, 64, 128	16
Epochs	30, 40, 50, 60, 70, 80, 90, 100	50
Learning rate	0.01, 0.001, 0.0001, 0.05, 0.005, 0.0005	0.005
Reduction ratio	4, 8, 16, 32	16
Weight decay	0.01, 0.001, 0.0001, 0.05, 0.005, 0.0005	0.0005

### Performance metrics of the networks

General performance metrics, including accuracy (Correct Classification Rate (CCR)), precision, sensitivity (recall or True Positive Rate (TPR)), specificity (True Negative Rate (TNR)), and F1-score, were used to evaluate each classification model. Accuracy is the number of samples that are correctly classified by the model divided by the total number of them (the sum of TPR and TNR divided by the sum of TPR, TNR, False Positive Rate (FPR), and False Negative Rate (FNR)). Sensitivity is the proportion of TPR outcomes over total actual positive cases (TPR divided by the sum of TPR and FNR). Specificity calculates the true proportion of all TNR outcomes (TNR divided by the sum of TNR and FPR). The precision is measured by TPR divided by the sum of TPR and FPR. The F1-score is computed as the weighted average of sensitivity and precision. We have a multi-class classification problem in this study. The One vs Rest technique is a common approach that faces each of the classes against the rest of them [63]. The performance metrics used in the ResNet18 and the proposed RCANet are expressed in Eqs (6)–(10). Where *i* indicates class *i* and *K* is the total number of classes in each set of the dataset.

$$\begin{array}{c} Accuracy=\frac{1}{K}\sum_{i=1}^{K}\frac{TPR_{i}+TNR_{i}}{TPR_{i}+TNR_{i}+FPR_{i}+FNR_{i}} \end{array}$$ (6)

$$\begin{array}{c} Precision=\frac{1}{K}\sum_{i=1}^{K}\frac{TPR_{i}}{TPR_{i}+FPR_{i}} \end{array}$$ (7)

$$\begin{array}{c} Sensitivity=\frac{1}{K}\sum_{i=1}^{K}\frac{TPR_{i}}{TPR_{i}+FNR_{i}} \end{array}$$ (8)

$$\begin{array}{c} Specificity=\frac{1}{K}\sum_{i=1}^{K}\frac{TNR_{i}}{TNR_{i}+FPR_{i}} \end{array}$$ (9)

$$\begin{array}{c} F1-score=[2\times \frac{Precision\times Sensitivity}{Precision_{i}+Sensitivity}] \end{array}$$ (10)

In the first set of experiments, the weighted performance metrics of the networks in the training, validation, and test steps are calculated and presented in Table 5. Also, the achieved weighted accuracy of each fold and the average weighted accuracy of the networks in the training and test steps in the second set of experiments are shown in Table 6. The values in brackets show the standard deviation. The low deviation between training and test values in Table 6 confirms the model’s robustness and rejects the probability of overfitting during the training step. Also, the low values in the standard deviations of the different measures prove the model’s stability. By comparing the obtained results of these tables found that the performance metrics of the proposed RCANet increased by 1% to 2% using the VDAB and the proposed RCANet achieves better performance metrics on the CPD than others in both experiments.

**Table 5:** The performance metrics of the networks on the training, validation, and test sets.

Step	Method	Dataset	Weighted Accuracy (%)	Weighted Precision (%)	Weighted Sensitivity (%)	Weighted Specificity (%)	Weighted F1-score (%)
Training	ResNet18	CPD	98.28	98.16	98	98.67	98.08
	RCANet		**99.34**	**99.39**	**98.89**	**98.99**	**99.14**
	ResNet18	Pollen13K	97.74	98.16	98.82	98.79	98.49
	RCANet		**98.94**	**99.5**	**99.18**	**98.65**	**99.33**
	ResNet18	Pollen23E	97.09	97	97.13	98.22	97.05
	RCANet		**99.19**	**99.65**	**98.98**	**99.15**	**99.31**
Validation	ResNet18	CPD	97.04	96.92	97.31	97.13	97.1
	RCANet		**98.82**	**97.99**	**98.97**	**98.41**	**98.47**
	ResNet18	Pollen13K	96.25	96.78	96.35	97.15	96.56
	RCANet		**98.1**	**98.06**	**98.96**	**97.3**	**98.5**
	ResNet18	Pollen23E	96.17	96.73	95.2	95.39	95.95
	RCANet		**98.1**	**98.33**	**98.38**	**97.96**	**98.35**
Test	ResNet18	CPD	96.82	96.58	97.02	96.89	96.78
	RCANet		**98.71**	**98.53**	**98.86**	**98.37**	**98.69**
	ResNet18	Pollen13K	95.79	96.33	96.38	95.79	96.34
	RCANet		**97.46**	**97.89**	**98.15**	**97.42**	**97.83**
	ResNet18	Pollen23E	95.92	96.69	94.86	95.43	96.4
	RCANet		**97.93**	**98.14**	**98.36**	**97.51**	**98.24**

**Table 6:** The weighted accuracy of the networks on the datasets with 5-fold cross-validation.

Step	Method	Dataset	Weighted accuracy of each fold (%)					Average Weighted Accuracy (%)
			Fold-1	Fold-2	Fold-3	Fold-4	Fold-5	
Training	ResNet18	CPD	97.36	98.01	99.18	96.89	99.17	98.12 (±0.930)
	RCANet		**99.16**	**99.63**	**99.98**	**99.06**	**99.45**	**99.46 (±0.331)**
	ResNet18	Pollen13K	96.16	96.58	96.04	97.95	96.97	96.74 (±0.688)
	RCANet		**98.84**	**98.47**	**98.91**	**98.06**	**98.12**	**98.48 (±0.352)**
	ResNet18	Pollen23E	96.83	96.94	97.38	97.03	95.29	96.69 (±0.725)
	RCANet		**98.35**	**98.2**	**97.92**	**96.29**	**98.24**	**97.8 (±0.768)**
Test	ResNet18	CPD	95.25	96.99	97.16	95.49	97.12	96.4 (±0.847)
	RCANet		**98.13**	**98.12**	**98.37**	**97.87**	**97.99**	**98.09 (±0.166)**
	ResNet18	Pollen13K	95.09	95.49	94.01	95.87	95.18	95.13 (±0.622)
	RCANet		**96.82**	**96.65**	**96.89**	**95.1**	**96.41**	**96.37 (±0.658)**
	ResNet18	Pollen23E	94.81	95.77	95.35	94.94	93	94.77 (±0.948)
	RCANet		**96.14**	**96.19**	**96.38**	**95.99**	**96.02**	**96.14 (±0.139)**

### Ablation study

A collection of experiments in which components of AI systems are withdrawn or replaced to measure the impact of these on the performance of the system is called an ablation study. Thus, graceful degradation of the performance of an AI system is expected if a certain component is withdrawn. Numerous experiments were performed to verify the contribution of all components and configurations of the proposed method, which would provide clearer insights into the model’s performance and the effectiveness of the VDAB. Thus, the impact of the existence or absence of the VDAB and important hyperparameters in its architecture including different pooling types, kernel sizes (k), and values of reduction ratio (r) in improving the performance metrics of the proposed method are measured in several sets of experiments. The impact of the VDAB on the performance metrics of the proposed method is measured by removing it in the first set of experiments. Table 7 shows the evaluation metrics of the proposed method are reduced without the VDAB. Hence, it can be concluded that the VDAB plays a vital role in the performance improvement of the proposed method. The VDAB adds 174,234 parameters to the proposed RCANet which will increase its memory usage and complexity time. The speed of the ResNet18 is low which is the backbone of the proposed RCANet. Therefore, the RCANet isn’t suitable for real-time applications.

**Table 7:** Performance metrics and number of parameters of the ResNet18 and the RCANet.

Method	Weighted Accuracy (%)	Weighted Precision (%)	Weighted Sensitivity (%)	Weighted Specificity (%)	Weighted F1-score (%)	Number of parameters
ResNet18	96.82	96.58	97.02	96.89	96.78	11,209,802
RCANet	**98.71**	**98.53**	**98.86**	**98.37**	**98.69**	11,384,036

The selection of the pooling operation is made according to the data at hand and it is used in neural networks to decrease variance and computation complexity. AP and MP are two common types of pooling, and we can not say which one is better than the other. The roles of different pooling types, such as AP, MP, and a combination of them (AP+MP) on the performance metrics of the proposed RCANet are investigated in the next set of experiments. The pooling layers were replaced in the architecture of the VDAB and the experiments were performed. The results of the experiments performed using different pooling types are shown in Table 8. It can be observed that when using both poolings the proposed RCANet outperforms the others. AP method smooths out the images and sharp features may not be identified while MP selects the brighter pixels of the image and is suitable for extracting the edges of the objects. We can extract the features related to the properties of within and border of pollen grains as well using AP and MP, respectively. Thus, the performance of the proposed method is improved with both AP and MP.

**Table 8:** The results of the experiments performed using different pooling types.

Method	Weighted Accuracy (%)	Weighted Precision (%)	Weighted Sensitivity (%)	Weighted Specificity (%)	Weighted F1-score (%)
RCANet (AP)	98.59	98.24	98.52	98.17	98.37
RCANet (MP)	98.44	98.13	98.39	97.99	98.36
RCANet (AP+MP)	**98.71**	**98.53**	**98.86**	**98.37**	**98.69**

There are various kernel sizes for convolution layers and *k* = 3 is a kernel size that looks at very few pixels at once and exports small complex features. It is a popular choice among others for decreasing the dimensions of the image and capturing neighborhood information. We perform a set of experiments with different values of *k* in the convolution layer of the VDAB and see which one is the best. Table 9 presents the results of the experiments done using different sizes of *k*. It confirms that the proposed RCANet achieved higher performance metrics with *k* = 3 than others. The different levels of benefit features from input images are extracted with *k* = 3 because it is effective in capturing local patterns and structures within images.

**Table 9:** Experiments result according to different sizes of *k* in the convolution layer of the VDAB.

Method	Weighted Accuracy (%)	Weighted Precision (%)	Weighted Sensitivity (%)	Weighted Specificity (%)	Weighted F1-score (%)
RCANet (k = 1)	98.49	98.46	98.81	98.2	98.62
RCANet (k = 3)	**98.71**	**98.53**	**98.86**	**98.37**	**98.69**
RCANet (k = 5)	98.68	98.15	98.76	97.95	98.45
RCANet (k = 7)	98.35	98.05	98.73	97.98	98.39

In the next set of experiments, we evaluate various values of the *r* parameter in performance improvement of the proposed method. As we said before, the number of neurons in the hidden layer of the MLP of the VDAB is determined by dividing *C* by *r*. Since they are dependent on values of the *C* and *r* parameters, thus the best value of *r* is experimentally identified. Table 10 presents that higher improvement in the performance criteria of the proposed RCANet with *r* = 16 occurs compared to its other values.

**Table 10:** Experiments result according to different values of *r* in the MLP of the VDAB.

Method	Weighted Accuracy (%)	Weighted Precision (%)	Weighted Sensitivity (%)	Weighted Specificity (%)	Weighted F1-score (%)
RCANet (r = 4)	98.62	98.38	98.75	98.23	98.56
RCANet (r = 8)	98.67	98.41	98.8	98.36	98.6
RCANet (r = 16)	**98.71**	**98.53**	**98.86**	**98.37**	**98.69**
RCANet (r = 32)	98.68	98.46	98.79	98.34	98.62

With performing the sets of experiments the impact of the VDAB and its hyperparameters on the performance improvement of the proposed method are determined. As a result, the proposed RCANet achieves better performance metrics with the VDAB by both AP and MP, *K* = 3, and *r* = 16 in this study.

### Comparison with previous state-of-the-art methods

The performance metrics of the proposed method and previous state-of-the-art methods for classifying pollen grain microscopic images on the CPD, Pollen13K, and Pollen23E datasets are demonstrated in Table 11. It can be confirmed that the proposed method has obtained higher weighted accuracy and weighted F1-score values than previous methods on the datasets.

**Table 11:** Comparison of the proposed method with previous state-of-the-art methods on the datasets.

Study	Approach Type	Dataset	Year	Weighted Accuracy (%)	Weighted F1-score (%)
Tsiknakis et al. [35]	Deep features-based	CPD	2022	97.5	96.89
Mahmood et al. [36]			2023	98.33	98.39
Our study			2024	**98.71**	**98.69**
Battiato et al. [27]	Handcrafted features-based	Pollen13K	2020	86.58	85.66
Battiato et al. [30]	Deep features-based		2020	89.73	89.14
Gui et al. [33]			2021	97.29	97.26
Mahbod et al. [34]			2021	96.28	96.3
Our study			2024	**97.46**	**97.83**
Goncalves et al. [24]	Human vision	Pollen23E	2016	63.57	40.87
Goncalves et al. [24]	Handcrafted features-based		2016	68.57	53.66
Sevillano and Aznarte [28]	Deep features-based		2018	97.22	96.69
Da Silva Soares et al. [32]			2021	96.5	96.75
Mahmood et al. [36]			2023	97.39	97.66
Our study			2024	**97.93**	**98.24**

### Feature maps representation

**Fig 16:**
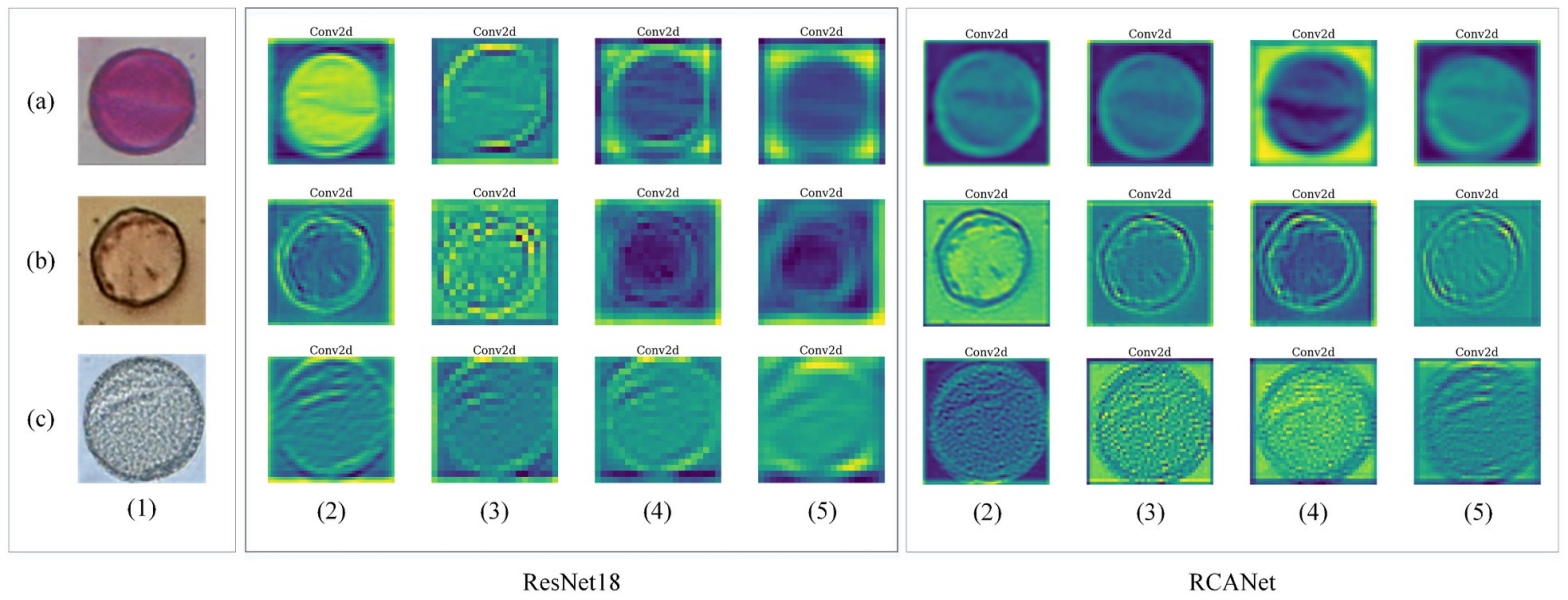
Input images from each dataset and their feature maps. ((a) is an image and its feature maps from the Satureja class of the CPD, (b) is an image and its feature maps from the Alnus (well-developed) class of the Pollen13K dataset, and (c) is an image and its feature maps from the Mabea class of the Pollen23E dataset, whereas (1) is input images, (5 ∼ 2) is the output feature maps of convolution layers of the middle Basic Blocks of the ResNet18 and the proposed RCANet, respectively.

We selected an example of an input image of each dataset and represented its feature maps in the output of convolution layers of the middle Basic Blocks of the ResNet18 and the proposed RCANet. The input images from each dataset and their feature maps are demonstrated in Fig 16.

**Fig 17:**
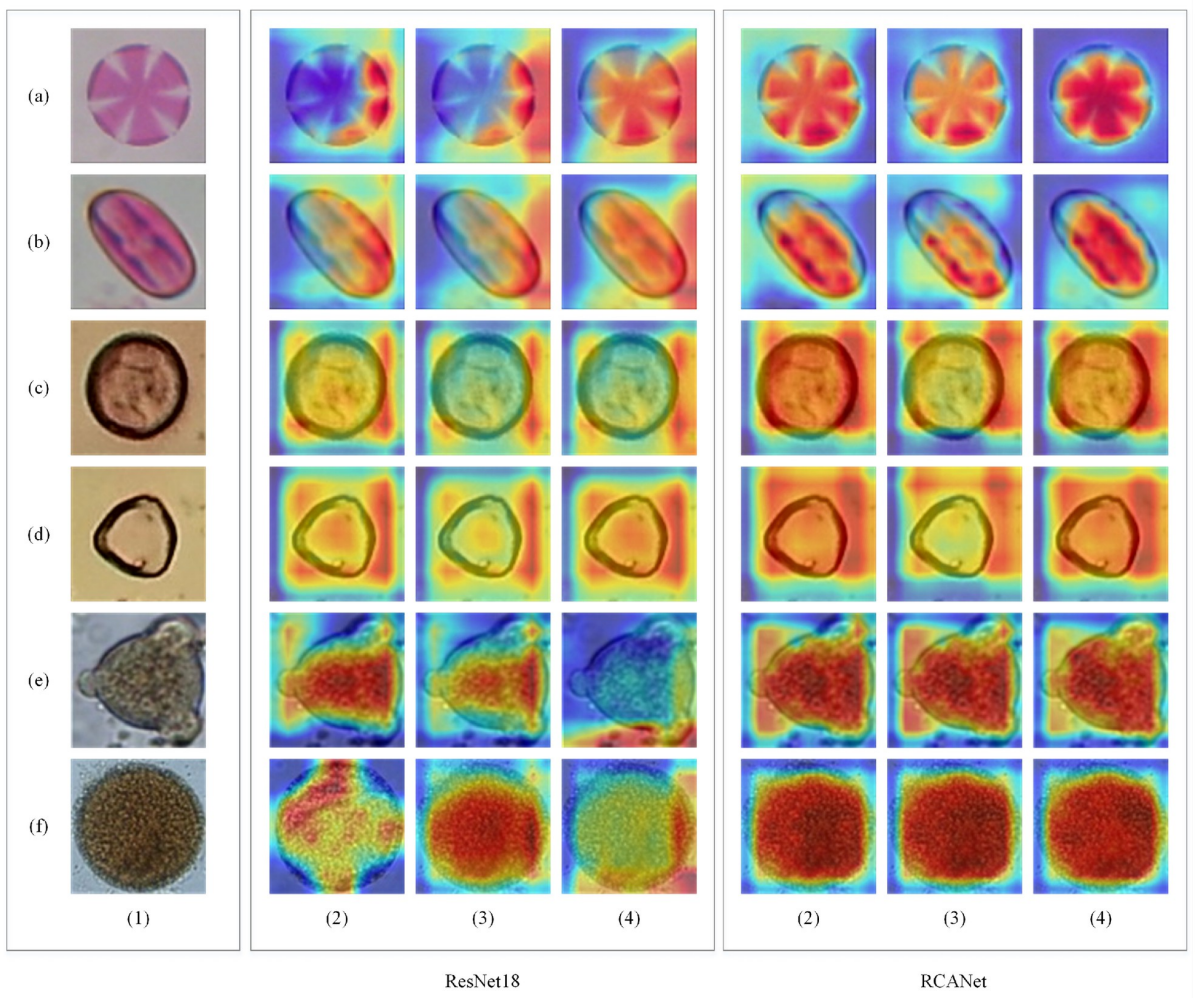
Grad-CAM, Grad-CAM++, and Score-CAM from the last Basic Block of the ResNet18 and the proposed RCANet on the datasets. (a) and (b) are Thymbra and Castanea of the CPD, (c) and (d) are Corylus avellana (well-developed) and Corylus avellana (anomalous) of the Pollen13K dataset, and (e), and (f) are Arrabidaea and Croton of the Pollen23E dataset, whereas (1) is input images, (2) includes Grad-CAM, (3) includes Grad-CAM++, and (4) includes Score-CAM images extracted from the ResNet18 and the proposed RCANet.

Gradient-weighted Class Activation Mapping (Grad-CAM) [64], Grad-CAM++ [65], and Score-CAM [66] are techniques to understand better image classification using gradients. We employed these approaches for visualizing the feature maps. The networks consider many features before deciding but do not describe considered specific features for prediction. We visualized discriminative regions in the image that were important for the prediction of the networks by Grad-CAM, Grad-CAM++, and Score-CAM. Some of the input images of the datasets and their corresponding Grad-CAM, Grad-CAM++, and Score-CAM from the last Basic Block of the ResNet18 and the proposed RCANet are shown in Fig 17, respectively.

#### The cases of correct and incorrect prediction

**Fig 18:**
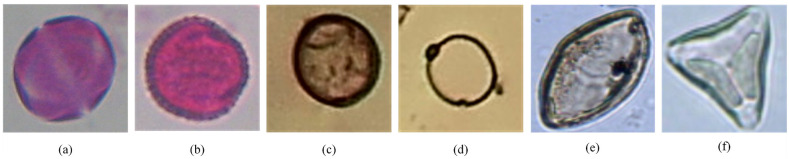
Samples of correct prediction of the three datasets. They categorize (a) Origanum and (b) Olea of the CPD, (c) Corylus avellana (well-developed) and (d) Debris of the Pollen13K dataset, and (e) Arecaceae and (f) Matayba of the Pollen23E dataset, respectively.

**Fig 19:**
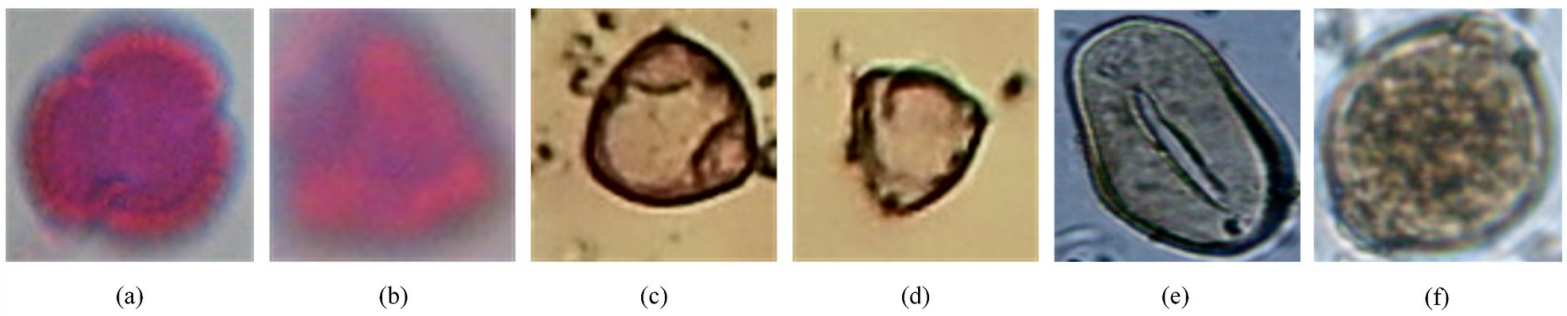
Samples of incorrect prediction of the three datasets. They categorize the cases that (a) Sinapis is misclassified as Olea and (b) Eucalyptus is misclassified as Ceratonia of the CPD, (c) Corylus avellana (well-developed) is misclassified as Alnus (well-developed) and (d) Corylus avellana (anomalous) is misclassified as Alnus (well-developed) of the Pollen13K dataset, and (e) Syagrus is misclassified as Arecaceae and (f) Dipteryx is misclassified as Arrabidaea of the Pollen23E dataset, respectively.

We display some images of the three datasets in Figs 18 and 19 that the proposed method has correctly and incorrectly categorized.

## Discussion

We analyzed the size of K in the VDAB, the performance metrics of the proposed method previous state-of-the-art methods, feature maps, and correct and incorrect predictions in this section.

### Analysis size of K in the VDAB

Woo et al. [55] and Park et al. [56] believed that using a large K in the architecture of the CBAM and BAM can help to obtain better performance matrices. Therefore, they employed standard 7 × 7 convolution and dilation layers in the structure of their channel-spatial attention module, respectively. We achieved better performance metrics with the standard 3 × 3 convolution layer of the VDAB based on Table 9. As a result, we found that the size of K in the convolution layer of each of the channel-spatial attention modules completely depended on the size and shape of RoI in the input images or feature maps.

### Analysis of the performance metrics

Classification accuracy improvement is one of the main challenges of pollen grain categorization, and many studies using deep learning have been done on it in recent years. Our study has been aimed at increasing accuracy and solving one of the existing challenges. Thus, we designed an attention block, inspired by the attention mechanism in biology, for using it in the architecture of CNNs to improve accuracy in the pollen grain classification problem. The VDAB first extracts the channels related to the shape of the pollen grain and then focuses on its spacial position. Thus, it helps the proposed RCANet to classify the pollen grain images with more accuracy, and the performance metrics of the proposed method are improved. Figs 15 and 16 confirm that the extracted feature maps by the proposed RCANet are better than the ResNet18 and it more pays attention to the structure and location of pollen grain than the ResNet18. Table 4 illustrates that the proposed RCANet successfully classified most of the samples and achieved better performance metrics such as accuracy than the ResNet18 in the training, validation, and test steps.

We used three different types of pollen grain datasets for training and evaluation of the proposed RCANet while all previous state-of-the-art methods (except study [36]) were learned and evaluated with only one pollen grain dataset. Therefore, the reported performance metrics are more comprehensive and reliable. The validation step is not performed in previous state-of-the-art methods but it is done in this study for the model’s generalization and avoiding its overfitting.

Comparison performance metrics of the proposed method including weighted accuracy and weighted F1-score with previous state-of-the-art methods on the datasets are shown the Table 9. It is observed that the proposed RCANet improved the weighted accuracy (by 38%, 17%, and 54%) and the weighted F1-score (by 3%, 57%, and 58%) on the CPD, Pollen13K, and Pollen23E than previous state-of-the-art methods, respectively. Although the proposed method obtained higher weighted accuracy and weighted F1-score than the previous related works, it has some limitations and weaknesses. The ResNet18 is in the backbone of the proposed RCANet and it has low speed. Also, the number of parameters, memory usage, and complexity time of the proposed RCANet is increased by embedding the VDAB in its architecture. Therefore, it is difficult to use the proposed method in the real-time applications.

### Feature maps analysis

We display some input images and their feature maps of the convolution layers of the middle Basic Blocks of the ResNet18 and the proposed method for evaluating the suggested attention block. Fig 16 shows that the RCANet better highlighted the features related to the shape of pollen grains than the ResNet18. This improvement has been achieved by helping the VDAB to the ResNet18. These feature maps increase the model performance. Fig 17 indicates that the proposed method pays more attention to the whole shape of the pollen grains of the input images and ignores other areas of them than the ResNet18. If the model pays more attention to the characteristics related to the pollen grain shape such as its borders, it will make a more correct decision-making about its class. The misclassification probability is increased by neglecting the structure of the pollen grains. The VDAB in the architecture of the RCANet causes more attention to the shape of pollen grains and it correctly classifies more input samples. Therefore, the network will achieve higher performance metrics by improving decision-making.

### Analysis of correct and incorrect prediction

Although the proposed method has achieved relatively high-performance metrics, it could not correctly classify some samples. The amount of blurring on the images and the structural similarity of some samples of different classes are the main reasons for the error in the model prediction.

## Conclusion and future work

This study presents the proposed RCANet to classify pollen grain microscopic images. We designed the VDAB and it is embedded in the Basic blocks of ResNet18 to focus more on the structure and location of pollen grain by extracting related feature maps. The VDAB is composed of VAB and DAB. The VAB chooses the more important feature maps related to the shape of pollen grains, and the DAB focuses on their location. The VDAB extracts effective feature maps that help the model for better prediction. The proposed method was trained and evaluated on three pollen grain datasets including CPD, Pollen13K, and Pollen23E. We used different types of data augmentation techniques on the training data of the CPD and Pollen23E datasets to avoid overfitting. The classification results demonstrated that the proposed method obtained higher performance metrics than previous state-of-the-art works. For future work, we will improve the VDAB in such a way that it imposes much fewer parameters on the network. Also, we will employ the improved VDAB on other CNNs like MobileNet to overcome its limitations and use it in real-time applications. The proposed VDAB and method can be improved for implementation on other similar pollen grain datasets, and their classification results are compared with the proposed RCANet.
